# Accumulation of Astragalosides and Related Gene Expression in Different Organs of *Astragalus Membranaceus* Bge. var *Mongholicus* (Bge.)

**DOI:** 10.3390/molecules190810922

**Published:** 2014-07-25

**Authors:** Yeon Bok Kim, Aye Aye Thwe, Xiaohua Li, Pham Anh Tuan, Sanghyun Lee, Jong Won Lee, Mariadhas Valan Arasu, Naif Abdullah Al-Dhabi, Sang Un Park

**Affiliations:** 1 Department of Crop Science, Chungnam National University, 99 Daehak-Ro, Yuseong-gu, Daejeon 305-764, Korea; E-Mails: yeonbokkim@hanmail.net (Y.B.K.); thwe.ayeaye@gmail.com (A.A.T.); Lixiaohua2007@hotmail.com (X.L.); tuan_pham_6885@yahoo.com (P.A.T.); ljw5407@yahoo.co.kr (J.W.L); 2 Department of Integrative Plant Science, Chung-Ang University, Anseong 456-756, Korea; E-Mail: slee@cau.ac.kr; 3 Department of Botany and Microbiology, Addiriyah Chair for Environmental Studies, College of Science, King Saud University, P. O. Box 2455, Riyadh 11451, Saudi Arabia; E-Mails: mvalanarasu@gmail.com (M.V.A.); naldhabi@ksu.edu.sa (N.A.A.)

**Keywords:** *Astragalus membranaceus*, astragalosides, gene expression, mevalonate pathway, triterpenoid saponin

## Abstract

*Astragalus membranaceus* is one of the most important traditional Korean and Chinese medicinal herbs because it contains triterpenoid saponins (astragaloside I, II, III, and IV), which have beneficial and pharmacological effects on health. In this study, we analyzed 10 mevalonate pathway genes that are involved in astragaloside biosynthesis using the Illumina/Solexa HiSeq2000 platform. We determined the expression levels of the 10 genes using quantitative real-time PCR, and analyzed the accumulation of astragalosides in different organs using high-performance liquid chromatography. Genes related to the mevalonate pathway were expressed in different levels in different organs. Almost all genes showed high transcript levels in the stem and leaf, with the lowest transcript levels being recorded in the root. In contrast, most astragalosides accumulated in the root. In particular, the astragaloside IV content was distributed in the following order: root (0.58 mg/g DW) > flower (0.27 mg/g DW) > stem (0.23 mg/g DW) > leaf (0.04 mg/g DW). In the root, astragaloside II exhibited the highest content (2.09 mg/g DW) compared to astragaloside I, III, and IV. Notably, gene expression did not follow the same pattern as astragaloside accumulation. We suggest carefully that astragalosides are synthesized in the leaves and stem and then translocated to the root. This study contributes towards improving our understanding of astragaloside biosynthesis in *A. membranaceus.*

## 1. Introduction

*Astragalus membranaceus* (Fisch.) Bge. (also termed *Astragalus mongholicus* Bge.) is commonly named the membranous milk-vetch root (English), huang qi (Chinese), ogi (Japanese), and hwanggi (Korean). It is one of the most important traditional Korean and Chinese medicinal herbs. *A. membranaceus* is a perennial flowering plant that is mainly distributed in the cool arid continental regions of the Northern Hemisphere and South America, with this genus being particularly diverse in southwestern Asia [1]. The genus *Astragalus* contains over 2,000 species distributed worldwide, with more than 250 species belonging to the angiosperm family Fabaceae [1]. The root of *A. membranaceus* is used to increase metabolism and digestion, to enhance the immune system, and to promote the healing of wounds and injuries. The root of *A. membranaceus* has been also used as a supplementary medicine during cancer therapy and has antiperspirant, antibacterial, antiviral, antioxidant, and anti-inflammatory properties [2,3].

**Scheme 1:**
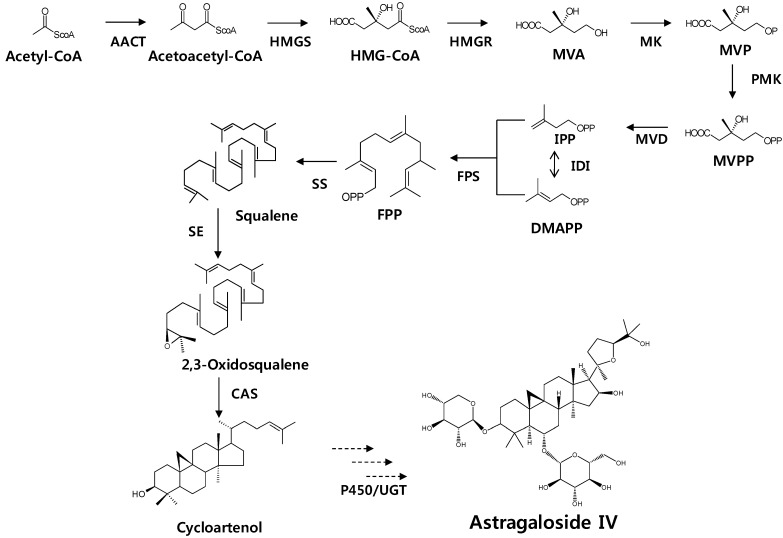
Astragaloside biosynthetic pathway. AACT, acetoacetyl-coenzyme A (CoA) thiolase; CAS, cycloartenol synthase; FPS, farnesyl diphosphate synthase; HMGR, 3-hydroxy-3-methylglutaryl coenzyme A (HMG-CoA) reductase; HMGS, HMG-CoA synthase; IDI, iso-pentenyl diphosphate isomerase; MVD, mevalonate diphosphate decarboxylase; MK, mevalonate kinase; PMK, phosphomevalonate kinase; SE, squalene epoxidase ; SS, squalene synthase; GT, glyosyltransferase; MVA, mevalonic acid; MVP, 5'-phosphomevalonate; MVPP, 5'-diphosphomevalonate; IPP, isopentenyl diphosphate; DMAPP, dimethylallyl diphosphate; FPP, Farnesyl diphosphate.

**Figure 1:**
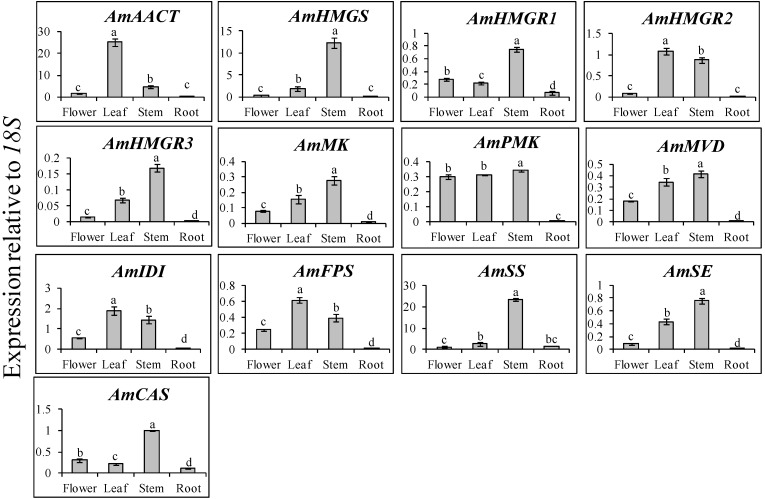
Transcript levels of triterpenoid biosynthetic genes in the flower, leaf, stem, and root of Huangqi. The height of each bar and the error bars show the mean and standard error, respectively, based on 3 independent measurements. Mean values indicated by the same letter are not significantly different at *p* ≤ 0.05, using Duncan Multiple Range Test (*n* = 3).

The terpenoids (also called isoprenoids) are one of the largest groups of natural products found in nature, with over 30,000 known examples, and their numbers continue to steadily grow [4]. Triterpenes are a large group of compounds that are arranged in a 4- or 5-ring configuration of 30 carbon molecules, with several oxygen molecules attached. Triterpenes are assembled from a C5 isoprene unit through the cytosolic mevalonate pathway [5] (Figure 1), to form a C30 compound, and are steroidal in nature. Triterpenoid saponins are triterpenes that belong to the group of saponin compounds. Phosphomevalonate kinase (PMK) catalyzes the phosphorylation of 5-phosphomevalonate into 5-diphosphomevalonate, which is an essential step in isoprenoid biosynthesis via the mevalonate pathway. Triterpenoid saponins are synthesized by farnesyl diphosphate (FPP) via the isoprenoid pathway. FFP is derived from isoprenyl diphosphate (IPP) and dimethylallyl diphosphate (DMAPP), in a reaction catalyzed by farnesyl diphosphate synthase (FPS) [6]. FPP is a precursor for squalene synthesis by squalene synthase (SS) in the first committed step toward sterol and triterpenoid biosynthesis [7,8]. Squalene is oxidized into 2,3-oxidosqualene by squalene epoxidase (SE), leading to the cyclization of triterpenoid skeletons, such as oleanane, ursane, lupeol, and dammarane [9]. Cycloartenol is an important membrane constituent that can serve as precursors to steroid hormones [10]. As shown in Scheme 1, it is formed from (*S*)-squalene-2, 3-epoxide by a cyclization reaction catalyzed by cycloartenol synthase. Finally, astragaloside might be synthesized by P450 or glycosyltransferase.

The *A. membranaceus* root contains various biologically active constituents, such as saponins, isoflavonoids, polysaccharides, and astragalosides [11,12]. Among these constituents, astragalosides form a class of cycloartane triterpenoid type glycosides that indicate root quality [13,14,15,16]. Astragalosides I, II, III, and IV are the major astragalosides in the *A*. *membranaceus* root.Astragalosides have various therapeutic effects and are used clinically in the treatment of diabetes and cardiovascular disease [17,18,19]. In particular, it has been reported that astragaloside IV has various beneficial effects on a range of processes, such as osteogenesis, angiogenesis, and metabolic syndrome, in addition to having neuroprotective, anti-inflammatory, and cardioprotective functions [13,14,15,16]. Furthermore, it has both healing and antiscarring properties in the natural treatment of wounds [20].

To date, many studies have been published about the astragalosides of *A. membranaceus.* However, research about the genes involved in astragaloside biosynthesis has not been reported for this species. Therefore, here, we investigated the expression levels of genes (*AmAACT*, *AmHMGS*, *AmHMGR*, *AmMK*, *AmPMK*, *AmMVD*, *AmFPS*, *AmSE*, and *AmCAS*) related to astragalosides biosynthesis, and analyzed the astragaloside content of different organs (e.g., flower, leaf, stem, and root) using quantitative real-time PCR (qRT-PCR) and high-performance liquid chromatography (HPLC), respectively. Our results are expected to provide baseline information towards elucidating the mechanism of astragaloside biosynthesis in *A*. *membranaceus.*

## 2. Results and Discussion

### 2.1. Isolation and Sequence Analysis of 10 Terpenoid Genes from A. membranaceus

We obtained one partial genes (*AmIDI*) and nine full-length gene (*AmAACT*, *AmHMGS*, *AmHMGR*, *AmMK*, *AmPMK*, *AmMVD*, *AmFPS*, *AmSE*, and *AmCAS*) from next generation sequencing (NGS) data of the *A. membranaceus* hairy root. The DNA sequence and the amino acid (aa) of partial gene, isopentenyl diphosphate isomerase (AmIDI, GenBank Accession No. KF355965) was 756 bp and 252 aa, respectively. As shown in Table 1, the open reading frames (ORF) of acetoacetyl-coenzyme A (CoA) thiolase (AmAACT, KF355956), HMG-CoA synthase (AmHMGS, KF355957), 3-hydroxy-3-methylglutaryl coenzyme A (HMG-CoA) reductase (AmHMGR1, KF355958), AmHMGR2 (KF355959), AmHMGR3 (KF355960), mevalonate kinase (AmMK, KF355961), phosphomevalonate kinase (AmPMK, KF355962), mevalonate diphosphate decarboxylase (AmMVD, KF355964), farnesyl diphosphate synthase (AmFPS, KF355966), squalene epoxidase (AmSE, KJ010819), and cycloartenol synthase (AmCAS, KJ010820) were 1155, 1383, 1707, 1695, 1710, 1167, 1527, 1263, 1030, 1587, and 2298 bp in length, respectively.

**Table 1:** Sequence analysis of genes obtained from *A. membranaceus*.

Gene Name	Full-Length (bp)	ORF(bp)	Amino Acid Sequence (aa)	MW(kDa)	pI Value	PSORT(Prediction of Targeting Localization)
AmAACT	-	1155	384	39.48	6.03	cytoplasm
AmHMGS	2088	1383	460	50.90	5.88	microbody (peroxisome)
AmHMGR1	2283	1707	568	60.84	8.45	mitochondrial inner membrane
AmHMGR2	2255	1695	564	60.15	6.89	plasma membrane
AmHMGR3	1971	1710	569	61.01	8.12	plasma membrane
AmMK	1298	1167	388	41.04	5.48	endoplasmic reticulum (membrane)
AmPMK	-	1527	508	55.13	5.67	plasma membrane
AmMVD	-	1263	420	46.32	6.04	microbody (peroxisome)
AmIDI	-	756 (partial)	252	-	-	plasma membrane
AmFPS	1167	1030	342	39.29	5.22	cytoplasm
AmSE	2134	1587	528	57.41	8.92	endoplasmic reticulum (membrane)
AmCAS	2833	2298	765	87.64	6.41	microbody (peroxisome)

These ORFs encoded proteins with 384, 468, 568, 564, 569, 388, 508, 420, 342, 528, and 765 aa, respectively. As shown in Table 1, the highest MW of AmCAS was 87.94 kDa, while the highest pI value of AmHMGR1 was 8.45. From the BLAST analysis of several-deduced amino acid sequences, AmAACT shared 89%, 89%, 89%, 84%, 91%, and 86% identity with *Camellia chekiangoleosa* (AGH32909), *Vitis vinifera* (XP_002265690), *Camellia oleifera* (ADD10719), *Ricinus communis* (XP_002522876), *Medicago sativa* (ACX47470), *Nicotiana tabacum* (AAU95618), respectively. AmHMGS shared 89%, 88%, 89%, 85%, and 82% identity with *Glycine max* (XP_003538436), *Medicago truncatula* (XP_003611167), *Cicer arietinum* (XP_004511613), *Theobroma cacao* (EOY24602), *R. communis* (XP_002509692), respectively. AmHMGR1 shared 84%, 84%, 78%, and 75% identity with *C. arietinum* (XP_004512291), *M. truncatula* (XP_003612421), *G. max* (XP_003517117), and *Glycyrrhiza uralensis* (AEH58930), respectively. AmMK shared 92%, 86%, 90%, 91%, 74%, 76%, and 74% identity with *C. arietinum2* (XP_004494628), *G. max* (XP_003521712), *M. truncatula* (XP_003626302), *C. arietinum1* (XP_004494627), *S. lycopersicum* (XP_004230181), *H. brasiliensis* (AAL18925), and *F. vesca* (XP_004302439), respectively. AmMVD shared 94%, 92%, 83%, 84%, and 83% identity with *C. arietinum* (XP_004497159), *G. max* (XP_003555870), *C. roseus* (ADR65113), *R. communis* (XP_002521172), and *F. vesca* (XP_004307061), respectively. AmPMK shared 91%, 91%, 87%, 77%, and 78% identity with *C. arietinum* (XP_004502634), *M.* *truncatula* (XP_003602220), *G. max* (XP_003526704), *V. vinifera* (XP_002275808), and *F. vesca* (XP_004300184), respectively. As shown in Table 1, the transit peptide of each protein was predicted to target localized regions in various organelles. AmAACT and AmFPS were located in the cytoplasm and AmHMGR-2, -3, and AmSS were located in the plasma membrane. AmHMGS and AmCAS were predicted to target the peroxisome. In particular, AmMK and AmSE were located in the endoplasmic reticulum.

To date, the genes involved in astragaloside biosynthesis have not been reported for *A. membranaceus.* The results of this study indicate that the MVA pathway genes of *A. membranaceus* have high homology with related genes recorded in the National Center for Biotechnology Information (NCBI) (Supplemental Material Figure S2-S8). In case of AACT, two cystine and one histidine residue are important for catalytic activity [21,22]. Other MVA pathway genes of *A. membranaceus* (*i.e*., AmHMGS, AmHMGR, AmMK, AmMVD, and AmFPS) exhibited conserved regions and essential residues of catalytic activity. MEP pathway enzymes are encoded by nuclear genes and targeted towards plastids. In comparison, MVA pathway genes are synthesized in cytosol, while MVA-derived IPP is transported into the mitochondria for the biosynthesis of ubiquinone [23]. In the predicted of targeting localizations based on the PSORT program, only AmAACT and AmFPS were observed in the cytoplasm. To elucidate the evolutionary relationships, phylogenetic trees were created based on the MVA pathway gene sequences obtained from the GenBank (Supplemental Material Figure S9–S15).

Each gene involved in astragaloside biosynthesis had different targeting localization. The expression levels of *AmHMGS* and *AmSS* were higher in the stem compared to the other organs (Figure 1). It was found that *Bacopa monniera AACT* had the highest expression in the roots and petals, followed by the sepals, stem, leaf, and pedicel [24]. However, in this study, the expression levels of *AmAACT* were the highest in the leaf, followed by the stem, flower, and root. Recent studies have reported that AACT plays an important role in isoprenoid biosynthesis of *Arabidopsis* [25], *Salvia* [26], *Medicago* [27], and Brahmi [24]. HMGR plays an important role in isoprenoid biosynthesis, catalyzing the transformation of HMG-CoA to mevalonate, which is the common precursor for several classes of essential metabolites in many organisms [28,29].

### 2.2. Expression Levels of Triterpenoid Biosynthetic Genes in Different Organs of A. Membranaceus

Analysis of expression levels in different organs showed that the terpenoid biosynthetic genes were constitutively expressed in *A. membranaceus* (Figure 1). Most genes showed the highest expression levels in the stem, except for *AmAACT*, *AmHMGR2*, *AmFPS*, and *AmIDI*. Additionally, the expression levels exhibited significant difference. In contrast, the lowest expression levels were recorded in the root for all genes*.* *AmAACT*, which involved from the start of the triterpenoid saponins biosynthetic pathway, had the highest expression in the leaf, and the lowest expression in the root. In particular, *AmAACT* transcription in the leaf was 5.4-, 17.9-, and 69.8-fold higher compared to that of the stem, flower, and root, respectively. The expression patterns of *AmMK*, *AmMVD*, and *AmSE* were fairly similar to those of *AmHMGR3*, with the highest levels occurring in the stem, followed by the flower and leaf, and finally the root. Among the various genes, *AmAACT* and *AmSS* exhibited the highest expression levels relative to *18S*, while *AmHMGR3* exhibited the lowest expression levels relative to *18S*. *AmPMK* showed similar expression levels in the flower, leaf, and stem. In contrast, *AmHMGS* and *AmSS* exhibited 6.7 and 8.9 times higher expression levels in the stem compared to the leaf. The qRT-PCR results of this study indicated that the expression levels of the first gene (*AmAACT*) involved in the mevalonate pathway were the highest in the leaf, whereas the expression levels of the gene (*AmCAS*) involved at the end of the pathway were the highest in the stem. 

### 2.3. Analyses of Astragalosides in Different Organs of A. Membranaceus

**Figure 2:**
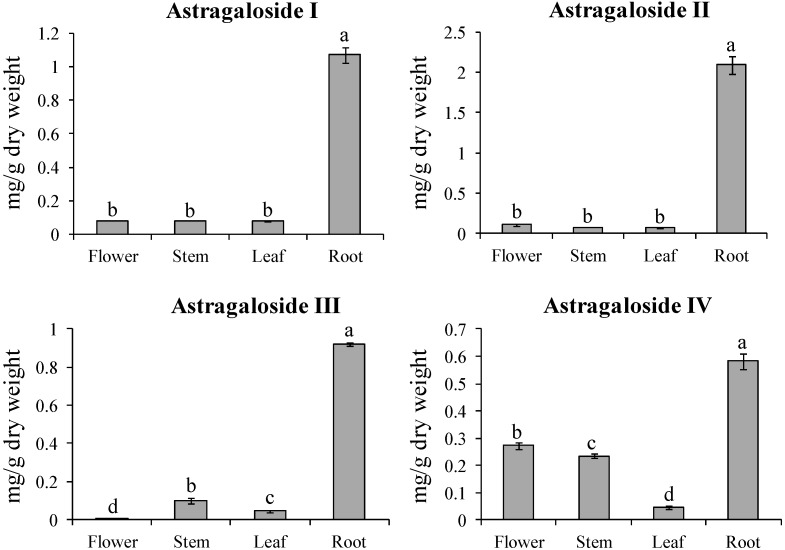
Astragaloside I, II, III, and IV content in the flower, stem, leaf, and root of Huangqi. The height of each bar and the error bars show the mean and standard error, respectively, based on 3 independent measurements.

The quantities of astragaloside I, II, III, and IV in the various organs of *A. membranaceus* were analyzed by HPLC (Figure 2; Supplemental Material Figure S1). Most astragalosides were accumulated in the root. Astragaloside I and II content in the root was 13.3 and 28.6 times higher compared to the leaf, while other organs showed similar content (0.07–0.11 mg/g dry weight [DW]). Like astragaloside I and II, the astragaloside III and IV content was 23- and 14.5-fold higher in the root compared to the leaf. The flower contained higher astragaloside IV content compared to astragaloside I, II, and III. In particular, astragaloside IV content was distributed in the following order: root (0.58 mg/g DW) > flower (0.27 mg/g DW) > stem (0.23 mg/g DW) > leaf (0.04 mg/g DW). The root contained higher astragaloside II content (2.09 mg/g DW) compared to astragaloside I, III, and IV. The astragaloside content displayed significant difference. *A. membranaceus* is an important medicinal plant in China and Korea. Recently, pharmacological research on *A. membranaceus* has focused on its immune-stimulating polysaccharides and other active ingredients that are useful in treating immune deficiency conditions [30]. Consequently, many studies have been involved in researching astragaloside IV [31,32,33,34,35]. Recently, it was described that telomerase activator TA-65 obtained from *A. membranaceus* elongates short telomeres and increases the health span of adult⁄old mice without increasing the incidence of cancer [36].

In general, there are several copies of the *HMGR* gene in prokaryotes and eukaryotes. HMGR in *A. membranaceus* had also three copies. The transcription of each HMGR gene was constitutively expressed in the different organs of *A. membranaceus*. *AmHMGR1* and *AmHMGR3* were more highly expressed in stem than in flower, leaf, and root, whereas *AmHMGR2* was more highly expressed in the leaf compared to other organs (Figure 2). Unexpectedly, gene expression from the first to last genes (*i.e*., *AmAACT* to *AmCAS*) of the triterpenoid saponins biosynthesis pathway was highly expressed in the leaf and stem only. Greater quantities of astragalosides were accumulated in the root compared to the other organs. It was reported that the genes for HbAACT1, HbHMGS1, HbHMGR1, HbMVK, HbPMK, and HbMVD are key enzymes in the natural biosynthesis of the rubber tree *Hevea brasiliensis* [37]. Natural products are often transported to a site of accumulation from a site of synthesis [38]. In *Arabidopsis*, cytokinin, caffeine, and nicotine are produced in roots and translocated to shoots [39]. Additionally, it was described that glucosinolates were synthesized in leaves and translocated to seed during development in *Tropaeolus majus* [40]. Moreover, Chen and Halkier [41] pointed out that a carrier-mediated uptake system had high specificity for glucosinolates by glucosinolates degradation products, amino acids, and sugars.

Very recently, we reported that expression levels of all genes (phenylalanine ammonia lyase, cinnamate 4-hydroxylase, 4-coumaroyl:CoA ligase, chalcone synthase, chalcone reductase, chalcone isomerase, isoflavone synthase, isoflavone O-methyltransferase, isoflavone 3′-hydroxylase and UDP-glucose: calycosin-7-O-β-d-glucosyltransferase) involved in calycosin and calycosin-7-O-β-d-glucoside biosynthesis in *A. membranaceus* were the highest in the flower, whereas calycosin and calycosin-7-O-β-d-glucoside content were the highest in the leaf and root, respectively. We suggested that increase of calycosin-7-O-β-d-glucoside in the roots might originate from high calycosin accumulation in the stem and leaf [42]. Similarly, in this study, all genes involved in triterpenoid pathway showed high transcript levels in leaf and stem, whereas astragalosides content was the highest in the root. The expression pattern of genes involved in astragaloside biosynthesis were did not match the accumulation pattern of astragalosides in different organs of *A. membranaceus*. Therefore, we presume carefully that astragalosides are synthesized in leaf and stem and then translocated into the root. Alternatively, glycosyltransferases (GTs) and P450 genes might regulate astragaloside biosynthesis after cycloartenol synthesis.

## 3. Experimental Section

### 3.1. Plant Materials and Growth Conditions

The seeds of Huangqi (*A. membranaceus* Bge. var *mongholicus*) were purchased from the Asia Seed Co., Ltd (Seoul, Korea). *A. membranaceus* plants (with 12 plants in each pot filled with the perlite-mixed soil to reduce error variation) were cultured in the Chungnam National University, Daejeon, Korea, greenhouse (25 °C and 50% humidity). After eight months, each organ (e.g., roots, stems, leaves, and flowers) was collected from 12 plants. All samples were immediately frozen in liquid nitrogen, and ground with a mortar and pestle for RNA isolation and astragaloside analysis.

### 3.2. Total RNA Extraction and cDNA Preparation

Total RNA was extracted from the specified organs using the cetyltrimethylammonium bromide (CTAB) method [43], with minor modifications, combined with a total RNA extraction kit (Geneaid, Taipei, Taiwan). RNA quantity and quality, respectively were measured by a NanoVue Plus Spectrophotometer (GE Healthcare, Seoul, Korea), and assessed by running 1 µg RNA on 1.2% formaldehyde RNA agarose gel. First-strand cDNA was synthesized from 1 µg total RNA in a total volume of 20 µL, using a ReverTra Ace- α-Kit (Toyobo, Osaka, Japan) and the oligo (dT)_20_ primer, according to the manufacturer’s instructions.

### 3.3. Illumina Sequencing

For Illumina sequencing, mRNA was purified from total RNA using Sera-Mag Magnetic Oligo(dT) beads (Illumina, San Diego, CA, USA). cDNA synthesis, library construction, and DNA sequencing were performed using the Illumina/Solexa HiSeq2000 platform developed by Seeders Inc (Daejeon, Korea) (unpublished data). The resulting high-quality reads were then deposited in the Short Read Archive at NCBI with the accession number SRR923811. Reads obtained by an Illumina sequencer were filtered and *de novo* was assembled by Velvet and Oases at high k-mers of 57 and 59 [44].

### 3.4. Quantitative Real-Time PCR Analysis

For qRT-PCR analysis, the TM Calculator program (http://bioinfo.ut.ee/primer3-0.4.0/) [45] was used to compute the PCR annealing temperatures. Real-time PCR assays were carried out in a total volume of 20 µL, containing 10 µL of 2× SYBR Green Real time PCR master mix (Toyobo), 0.5 µM (each) of specific primers, and 5 µL of cDNA diluted 20-fold. Thermal cycling conditions were as follows: 95 °C for 3 min, followed by 40 cycles at 95 °C for 15 s, 72 °C for 20 s, and at an annealing temperature of 55 °C for 30 s. PCR products were analyzed by Bio-Rad CFX Manager 2.0 software. The reaction was performed in triplicate. The expression of each gene was calculated by the method of relative quantification, using *Am18S* as the reference.

### 3.5. Bioinformatics Analysis

Sequence similarities were calculated by the Basic Local Alignment Search Tool (BLAST) (http://blast.ncbi.nlm.nih.gov/) [46]. The deduced amino acid sequences of each gene were aligned using BioEdit (Biological sequence alignment editor). Gene-specific primers were designed using an online program (https://www.genscript.com/ssl-bin/app/primer) (Table 2). Theoretical molecular weights (MW) and isoelectric point (p*I*) values were calculated by the Compute p*I/*Mw tool (http://ca.expasy.org/tools/pi_tool.html) [47] (Table 1). The putative target location of the plant was predicted online through PSORT (http://psort.hgc.jp/form.html) [48] (Table 1).

**Table 2:** Primers used in this study.

Primer Name	Sequence 5' → 3'	Size (bp)
*AmAACT-RT(F)*	GGTGAGCGGAGAGAAGGCAT	110
*AmAACT-RT(R)*	CGAGTGCTGGAGCGGTTGTA	
*AmHMGS-RT(F)*	CCTTCTTCGGCATTGCTTTCATC	127
*AmHMGS-RT(R)*	TCGAGATCCCGGCTTTGGTA	
*AmHMGR1-RT(F)*	CCTTCTTCGGCATTGCTTTCATC	180
*AmHMGR1-RT(R)*	ACTCCGGCAGTGGTTTCCTG	
*AmHMGR2-RT(F)*	GCCGGCCACCATAAACGA	155
*AmHMGR2-RT(R)*	CGACGGAGAAGAAGAGGGTGAA	
*AmHMGR3-RT(F)*	GCCGGCCACCATAAACGA	164
*AmHMGR3-RT(R)*	GGTCGGCAATTTTCGATGGTAG	
*AmMK-RT(F)*	AACATGCCGTTGTTCACGGA	139
*AmMK-RT(R)*	AACTCCAATGCCGCATCGTT	
*AmPMK-RT(F)*	AGATCACCCGGACAGGAAGGA	142
*AmPMK-RT(R)*	CCGCACATAGCGATGACTTCC	
*AmMVD-RT(F)*	TAAGGGAGATCCGCGCTCGT	144
*AmMVD-RT(R)*	CAGCTGACGAAGCCAGTCCA	
*AmIDI-RT(F)*	TGCTGGTGAGGGAGGTTTGAA	116
*AmIDI-RT(R)*	TCATGTCAGCGACCTCACCAA	
*AmFPS-RT(F)*	CGACCGGATGCTGGACTACA	136
*AmFPS-RT(R)*	CCAACCAAGAGCACTGGCAA	
*AmSS-RT(F)*	AAGCAGATCCCTCCGGAACC	113
*AmSS-RT(R)*	ACAGCGTTGCGAAGTTCGGT	
*AmSE-RT(F)*	TGGAACAAGGAACCGTGACATCT	150
*AmSE-RT(R)*	ACAAAGAGAACGCCTCAAGTTGGA	
*AmCAS-RT(F)*	TGGAGATTTCCCACAGCAGGA	150
*AmCAS-RT(R)*	CAAGTTGCGGCATTTGGTGT	
*Am18S(F)*	TGCAGAATCCCGTGAACCATC	104
*Am18S(R)*	AGGCATCGGGCAACGATATG	

### 3.6. Astragaloside Analysis

Astragaloside analysis was carried out by HPLC [49]. Two hundred milligrams of powdered samples were extracted with 5 mL of 80% (v/v) ethanol at room temperature for 30 min. The samples were extracted three times, to quantify astragaloside levels. Then, the solvent was evaporated, and 1 mL of 80% methanol was added. Subsequently, the extracts were centrifuged, and the supernatant was filtered with a 0.45 μm Acrodisc syringe filter (Pall Corp., New York, NY, USA). HPLC analysis was performed with a C18 column (250 × 4.6 mm, 5 μm; RS tech). The mobile phase was a gradient prepared from mixtures of acetonitrile and 0.3% formic acid, and the column temperature was maintained at 30 °C. The flow rate was maintained at 1 mL/min. An Evaporative Light Scattering Detector (Futecs Co., Daejeon, Korea) was used for astragaloside analysis. The concentrations of the astragalosides were determined by using a standard curve. All samples were analyzed in triplicate.

### 3.7. Statistical Analysis

All statistical analyses were performed using the statistical analysis software SPSS 17.0 (SPSS Inc., Chicago, Illinois, USA, 2009). All data are given as the mean and standard deviation of triplicate experiments. The significant differences among means were determined by Duncan Multiple Range Test.

## 4. Conclusions

In this study, gene expression did not correspond with astragaloside accumulation in the various organs of *A. membranaceus*. Interestingly, high levels of astraglosides were detected in the roots and genes involved in triterpenoid saponin biosynthesis showed high expression levels in the leaf and stem. We presume carefully that astragalosides are synthesized in the leaves and stem and then translocated to the root. Alternatively, GTs and P450 genes might regulate astragaloside biosynthesis after cycloartenol synthesis. To explain adequately the mechanism of astragaloside biosynthesis and translocation in *A. membranaceus*, analysis of the protein expression level and enzymatic activity of mevalonate pathway gene in *A. membranaceus* should be examined in the near future.

## Supplementary Materials

Supplementary materials can be accessed at: http://www.mdpi.com/1420-3049/19/8/10922/s1.

## Supplementary Files

Supplementary File 1
