# Supplementary material for: Accumulation of Astragalosides and Related Gene Expression in Different Organs of Astragalus Membranaceus Bge. var Mongholicus (Bge.)
Source: Molecules. 2014 Jul 25;19(8):10922–35. doi: 10.3390/molecules190810922 (PMC6270750; doi:10.3390/molecules190810922)

Supplemental Materials

Figure S1. (A) HPLC standards chromatogram of astragaloside IV (1), III (2), II (3) and I (4); (B) Typical HPLC chromatogram of astragaloside IV (1), III (2), II (3) and I (4) in the root of *A. membranaceus*.

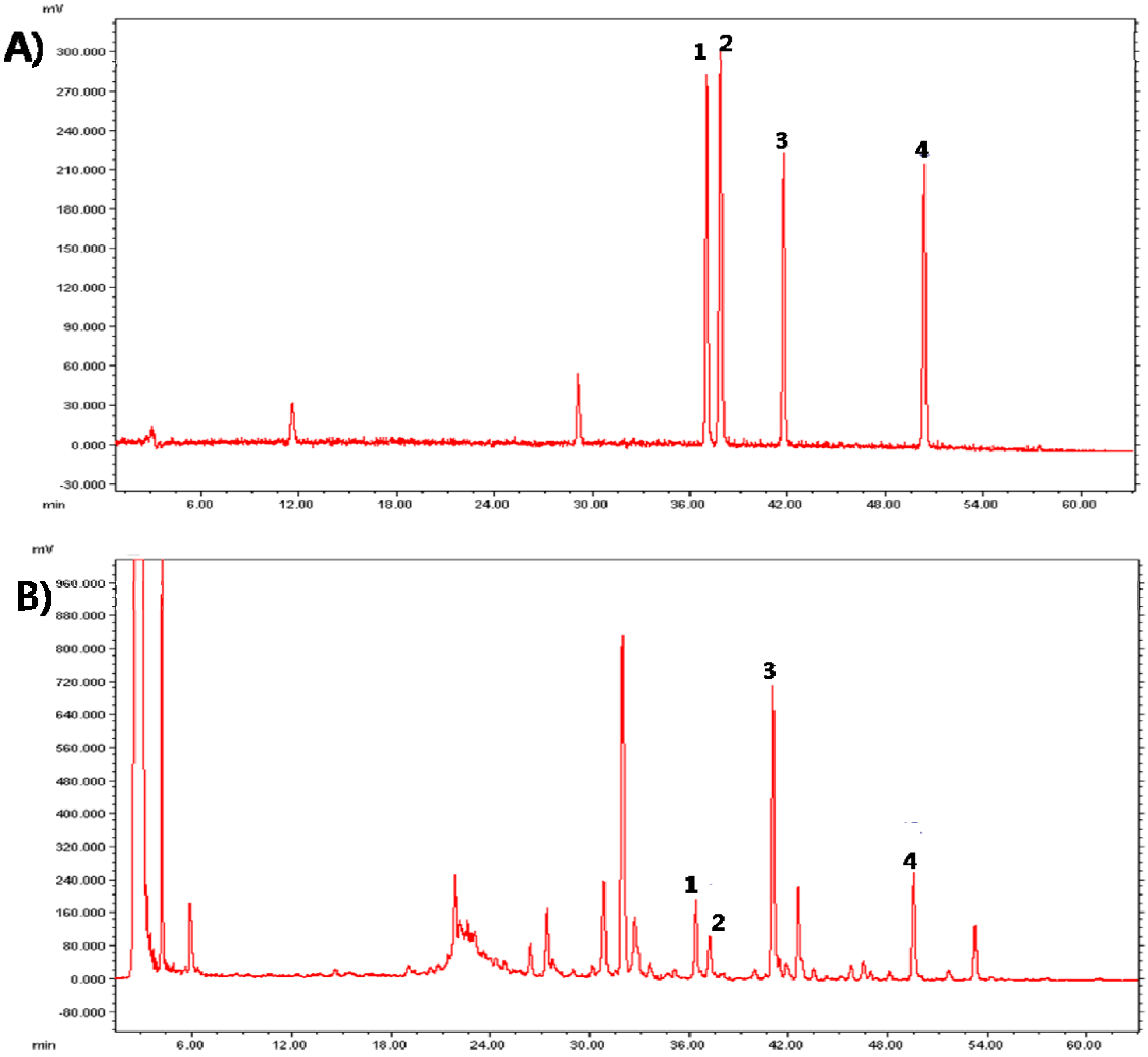

Figure S2. Multiple alignments of the amino acid sequences of AmAACT with other AACTs. Identical residues are indicated by large black boxes, and similar residues are indicated by small black boxes. Black stars indicate essential residues of catalytic activity. Alignment was generated using the BioEdit program. *C. chekiangoleosa* (AGH32909), *V. vinifera* (XP_002265690), *C. oleifera* (ADD10719), *R. communis* (XP_002522876), *A. membranaceus* (KF355959), *N. tabacum* (AAU95618), and *M. sativa* (ACX47470).

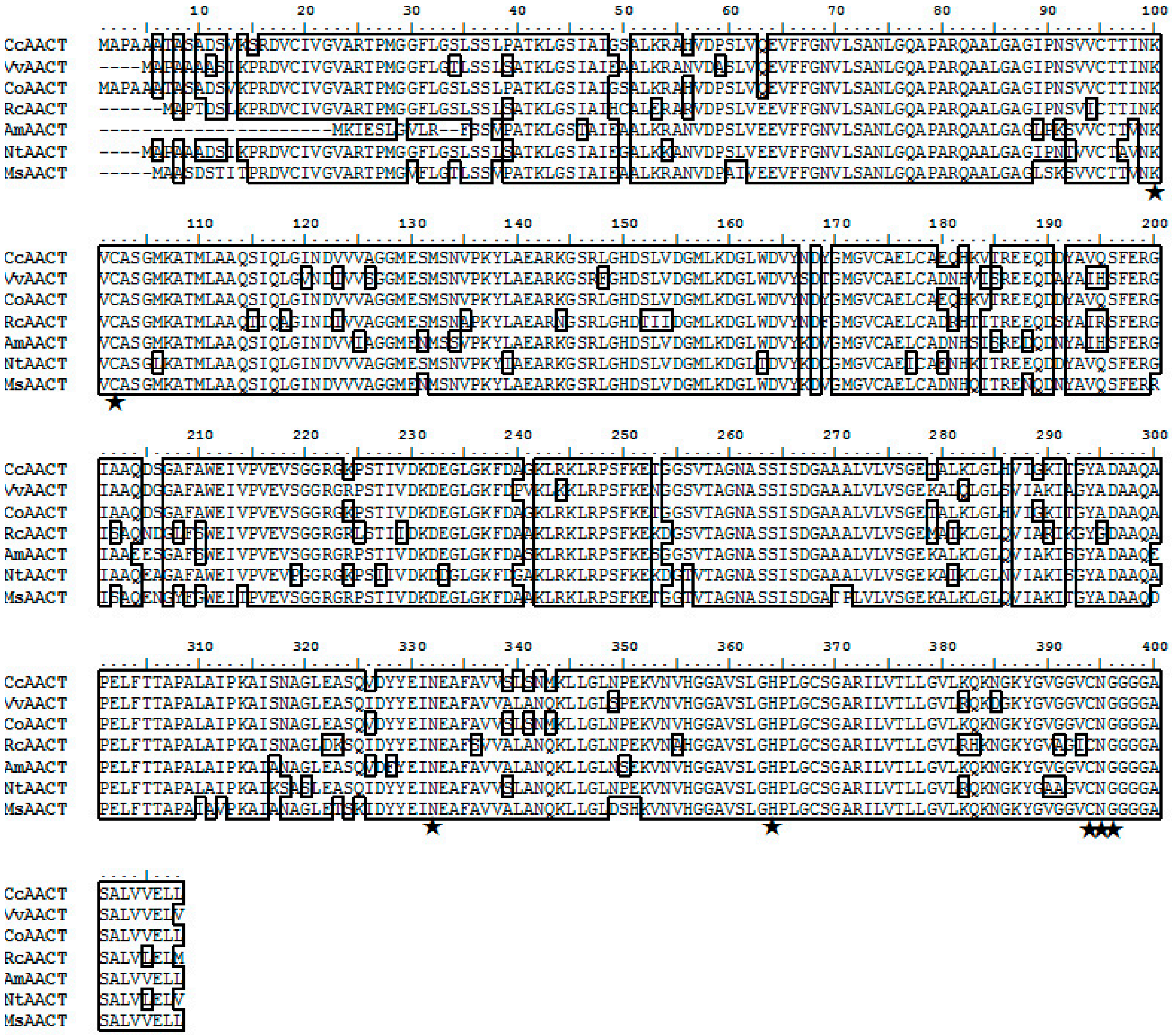

Figure S3. Multiple alignments of the amino acid sequences of AmHMGS with other HMGSs. Identical residues are indicated by large black boxes, and similar residues are indicated by small black boxes. Black stars indicate essential residues of catalytic activity. *A. membranaceus* (KF355957), *Glycine max* (XP_003538436), *Medicago truncatula* (XP_003611167), *Cicer arietinum* (XP_004511613), *Theobroma cacao* (EOY24602), and *R. communis* (XP_002509692).

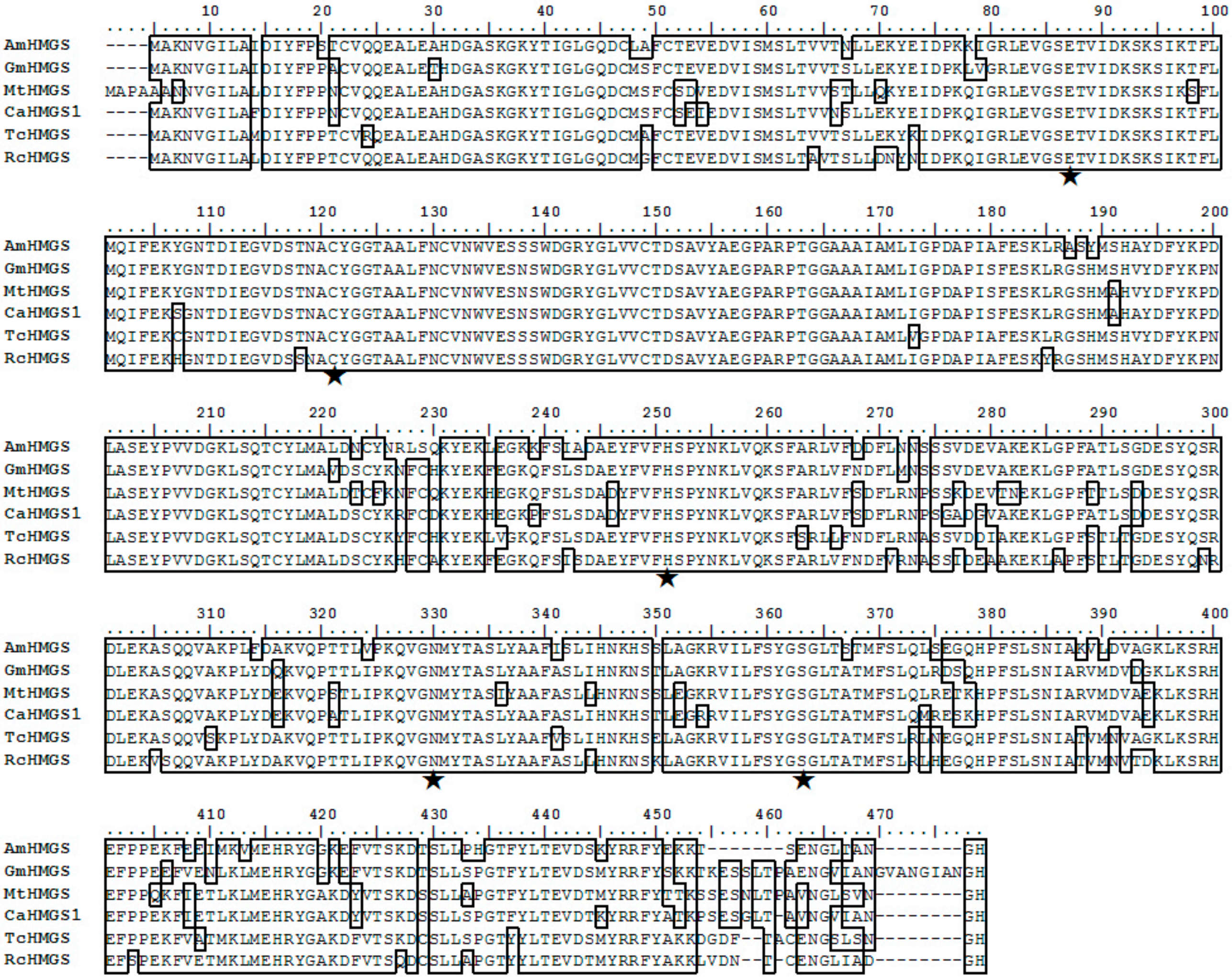

Figure S4. Multiple alignments of the amino acid sequences of AmHMGR with other HMGRs. Identical residues are indicated by large black boxes, and similar residues are indicated by samll black boxes. Black triangles indicate essential residues of catalytic activity. I, II, III, and IV indicate binding sites for HMG-CoA (I and II) and NADP(H)-binding (III and IV). Red boxes indicate highly conserved motifs. *A. membranaceus* (HMGR1, 2, and 3; KF355958, KF355959, and KF355960, respectively), *C. arietinum* (XP_004512291), *M. truncatula* (XP_003612421), *G. max* (XP_003517117), *Glycyrrhiza uralensis* (AEH58930), and *G. uralensis* (AEH58930).

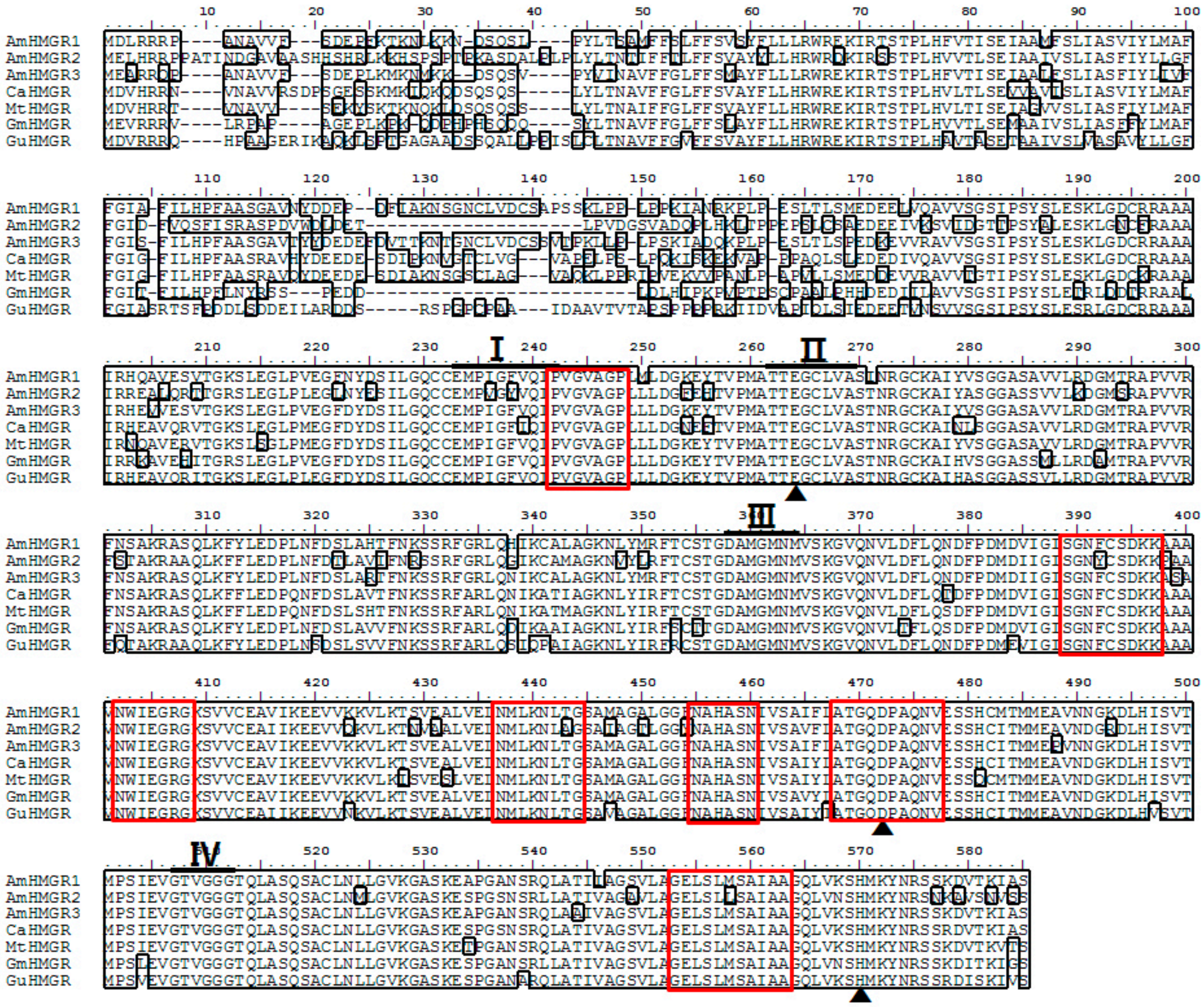

Figure S5. Multiple alignments of the amino acid sequences of AmMK with other MKs. Identical residues are indicated by large black boxes, and similar residues are indicated by small black boxes. Black triangles indicate essential residues of catalytic activity. Red boxes indicate highly conserved motifs. *A. membranaceus* (KF355961), *C. arietinum2* (XP_004494628), *G. max* (XP_003521712), *M. truncatula* (XP_003626302), *C. arietinum1* (XP_004494627), *S. lycopersicum* (XP_004230181), *H. brasiliensis* (AAL18925), and *F. vesca* (XP_004302439).

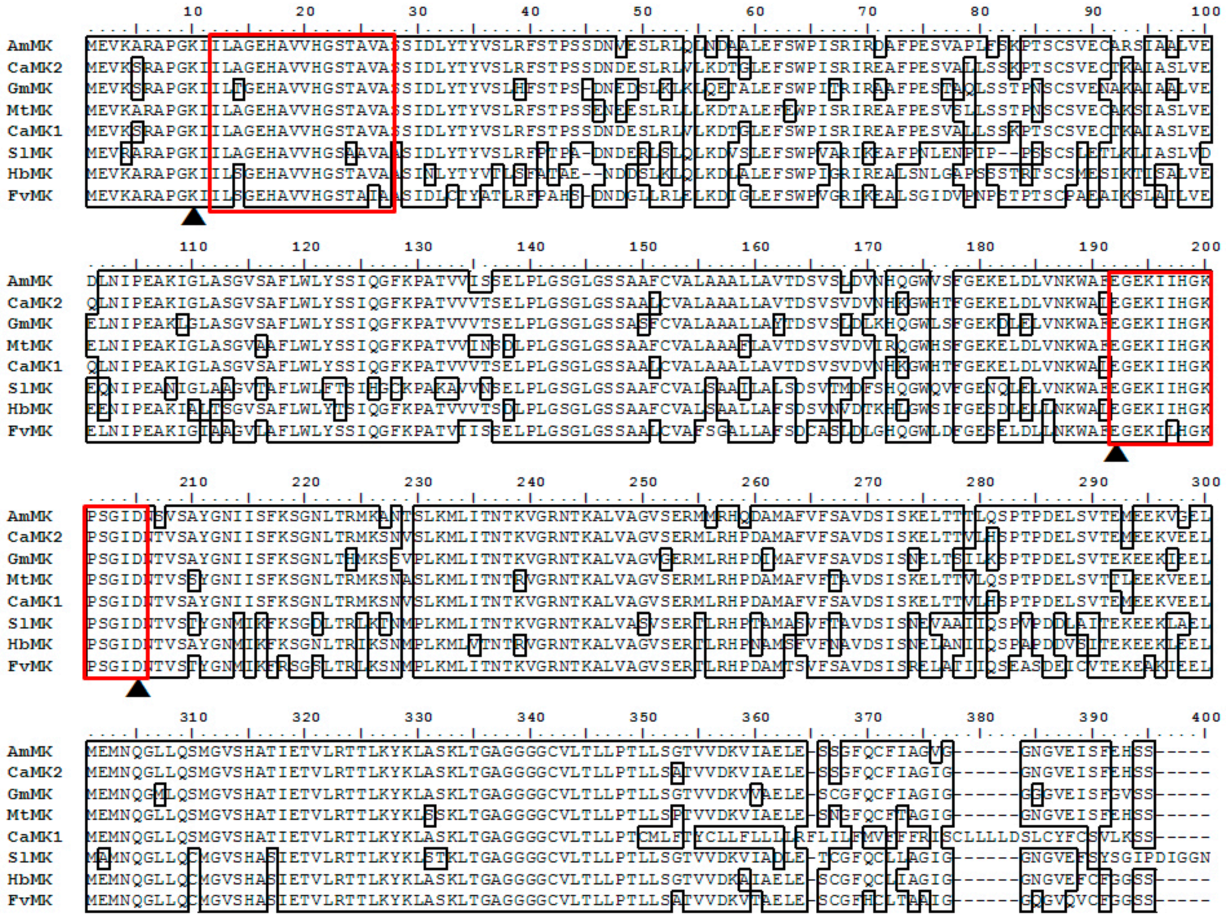

Figure S6. Multiple alignments of the amino acid sequences of AmPMK with other PMKs. Identical residues are indicated by large black boxes, and similar residues are indicated by small black boxes. *A. membranaceus* (KF355962), *C. arietinum* (XP_004502634), *M. truncatula* (XP_003602220), *G. max* (XP_003526704), *V. vinifera* (XP_002275808), and *F. vesca* (XP_004300184).

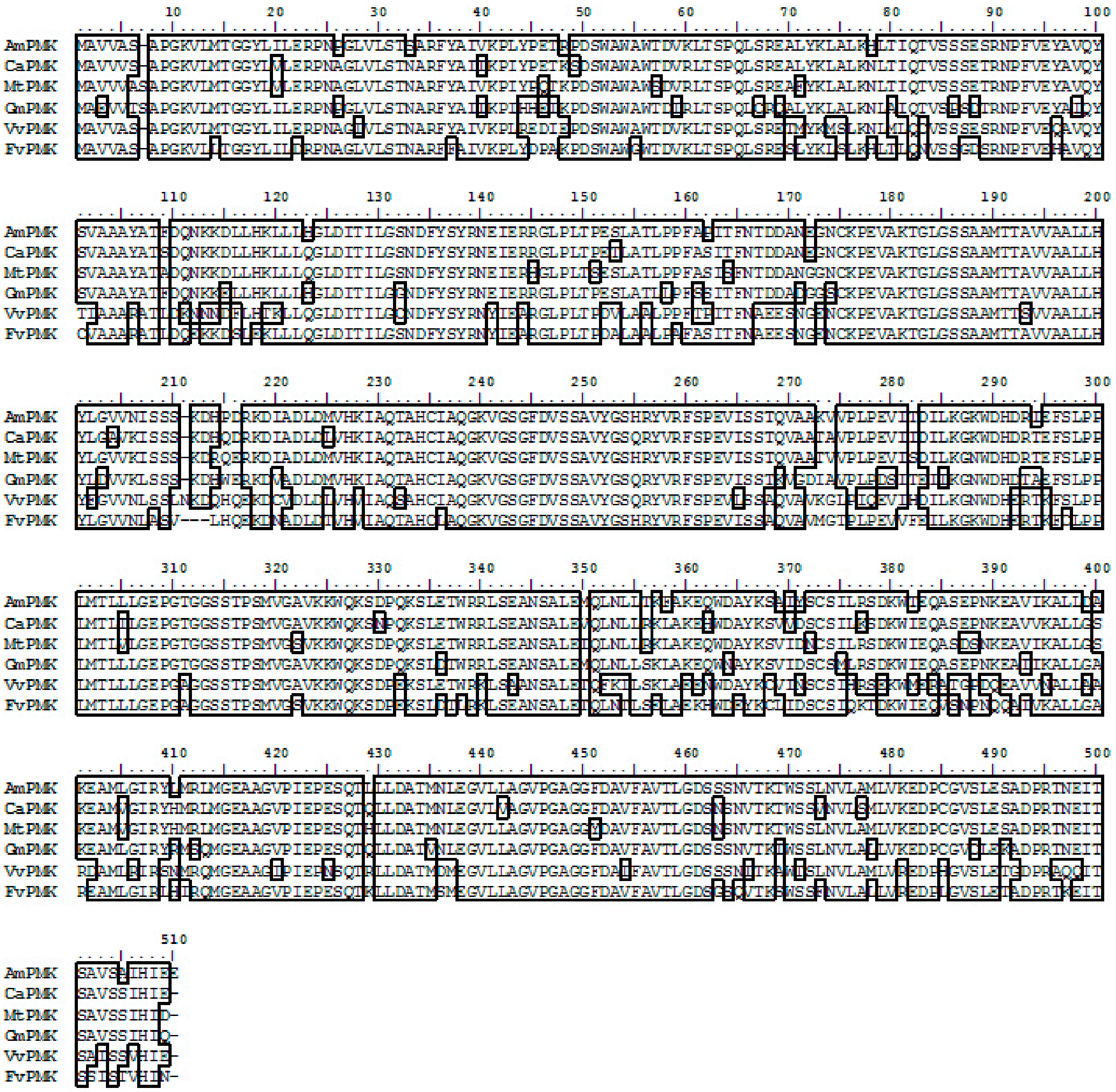

Figure S7. Multiple alignments of the amino acid sequences of AmMVD with other MVDs. Identical residues are indicated by large black boxes, and similar residues are indicated by small black boxes. Black stars indicate region flanking Lys 27 and Asp 314 are displayed. *A. membranaceus* (KF355964).*C. arietinum* (XP_004497159), *G. max* (XP_003555870), *C. roseus* (ADR65113), *R. communis* (XP_002521172), and *F. vesca* (XP_004307061),

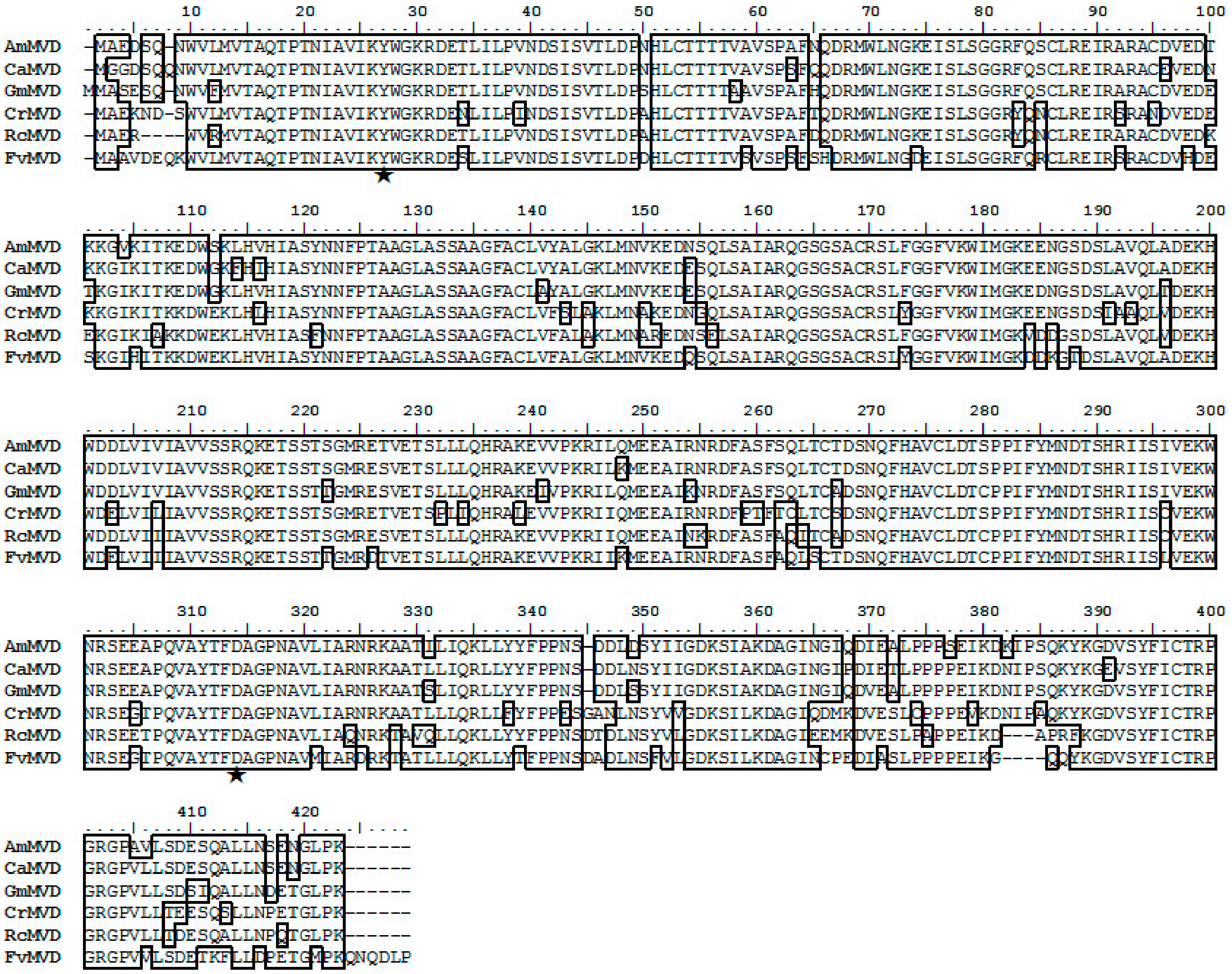

Figure S8. Multiple alignments of the amino acid sequences of AmFPS with other FPSs. Identical residues are indicated by large black boxes, and similar residues are indicated by small black boxes. Red boxes indicate two highly conserved regions of all trans-prenyl diphosphate synthases. Black stars indicate aromatic residues essential of the product length. *A. membranaceus* (KF355966), *G. uralensis* (ADE18770), *C. arietinum* (XP_004486372), *M. truncatula* (XP_003594327), *M. sativa* (ADC32809), and *G. max* (XP_003534984).

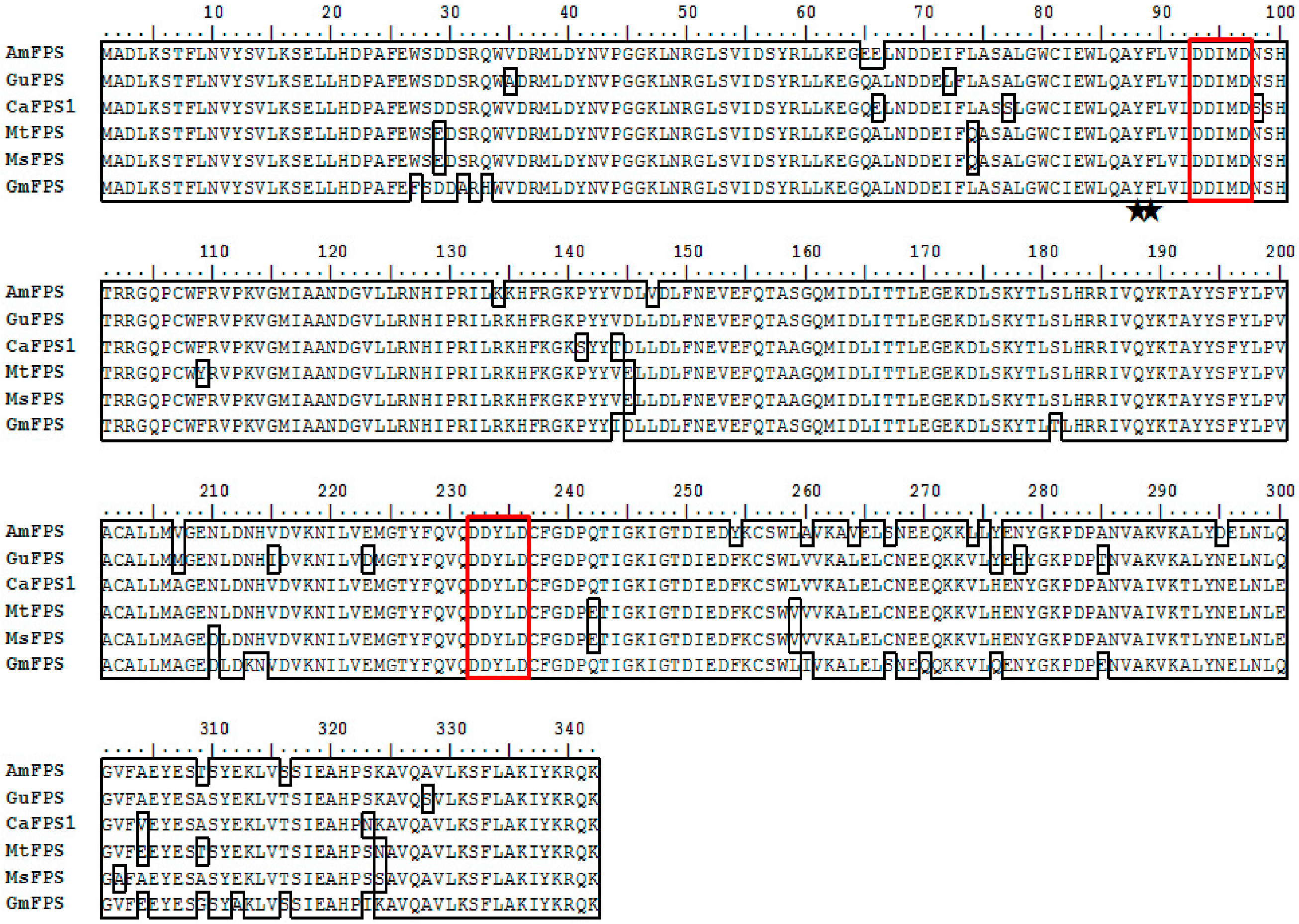

Figure S9. Phylogenetic tree of AACTs. *M. truncatula* (XP_003618040), *G. max* (XP_003545555), *C. arietinum* (XP_004491615), *S. lycopersicum* (XP_004243582), *H. brasiliensis* (BAF98276), *Bacopa monnieri* (ACU87560), *Camellia chekiangoleosa* (AGH32909), *V. vinifera* (XP_002265690), *C. oleifera* (ADD10719), *R.* ***communis*** (XP_002522876), *M. sativa* (ACX474), and *A. membranaceus* (KF35595670).

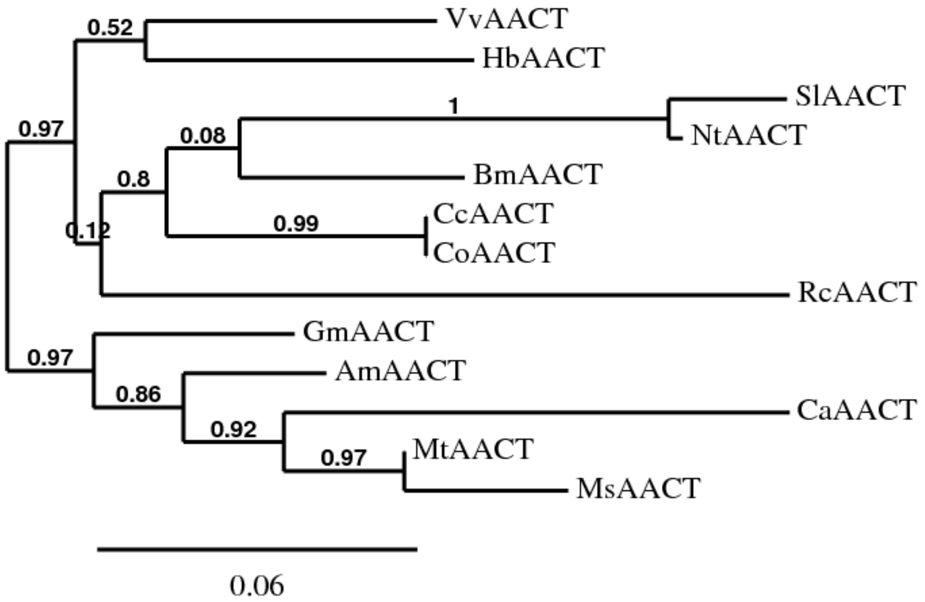

Figure S10. Phylogenetic tree of HMGSs. *G. max* (XP_003538436), *M. truncatula* (XP_003611167), *C. arietinum* (XP_004511613), *T.cacao* (EOY24602), *H. brasiliensis* (AAS46245), *R. communis* (XP_002509692), *V. vinifera* (XP_002282434), *Siraitia grosvenorii* (AEM42970), *Panax ginseng* (ADI80347), *C. roseus* (AEC13715), and *A. membranaceus* (KF355957).

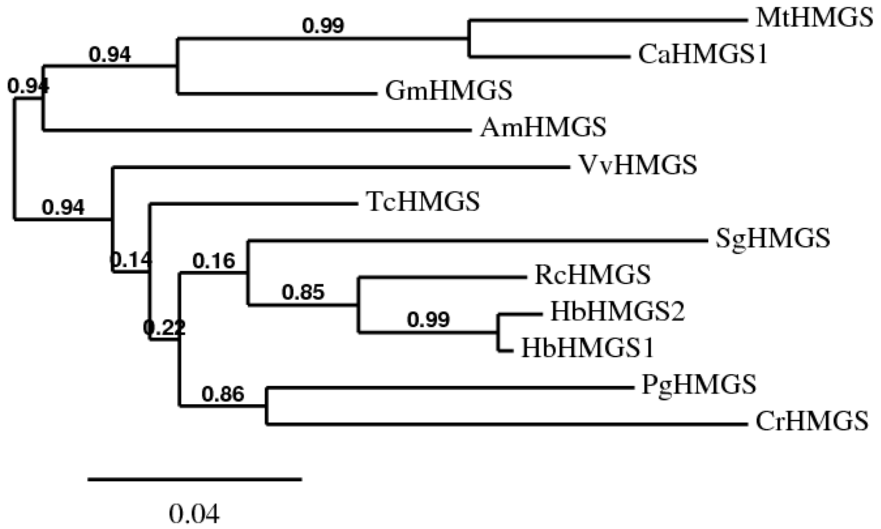

Figure S11. Phylogenetic tree of HMGRs. *C. arietinum* (XP_004512291), *M. truncatula* (XP_003612421), *G. max* (XP_003517117), *Glycyrrhiza uralensis* (AEH58930), *R. communis* (XP_002528521), *P. quinquefolius* (ACV65036), *Cucumis sativus* (XP_004139979), *S. chacoense* (AEX26934), *Solanum tuberosum* (AEX26933), *Fragaria vesca* (XP_004290088), *Morus alba* (AAD03789), *N. attenuate* (AAO85554), *N. tabacum* (AAB87727), *V. vinifera* (XP_002265638), and *Dimocarpus longan* (AET72044), *A. membranaceus* (HMGR1, 2, and 3; KF355958, KF355959, and KF355960, respectively).

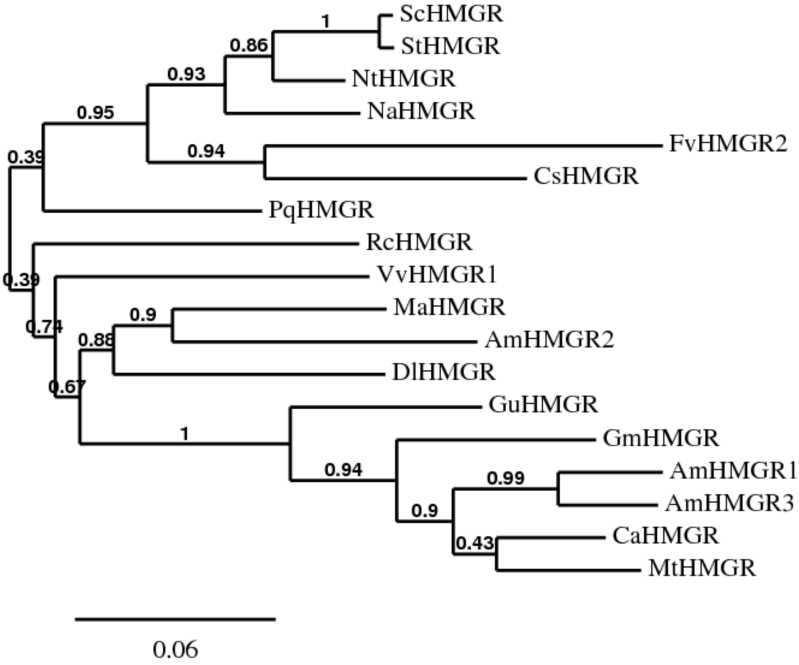

Figure S12. Phylogenetic tree of MKs. *C. arietinum2* (XP_004494628), *G. max* (XP_003521712), *M. truncatula* (XP_003626302), *C. arietinum* (XP_004494627), *S. lycopersicum* (XP_004230181), *H. brasiliensis* (AAL18925), *F. vesca* (XP_004302439), *N. sylvestris* (AGR67371), *Siraitia grosvenorii* (AEM42972), *Bacopa monnieri* (AFJ93086), *R. communis* (XP_002512365), *P. notoginseng* (AFN02124), and *A. membranaceus* KF355961).

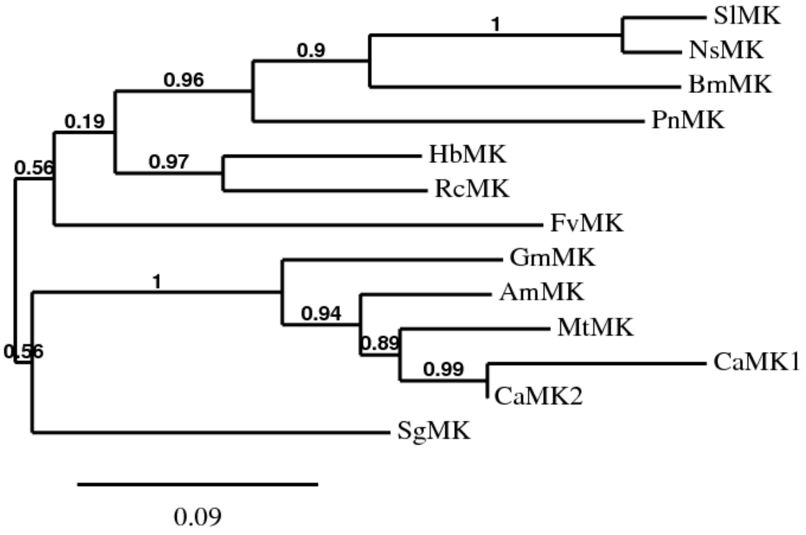

Figure S13. Phylogenetic tree of PMKs. *C. arietinum* (XP_004502634), *M. truncatula* (XP_003602220), *G. max* (XP_003526704), *V. vinifera* (XP_002275808), *F. vesca* (XP_004300184), *A. membranaceus* (KF355962), *T. cacao* (EOY25236), *S. lycopersicum* (XP_004241759), *C, sativus* (XP_004136210), *H. brasiliensis* (AAL18926), *S. grosvenorii* (AEM42973), and *Arnebia euchroma* (ABY27562).

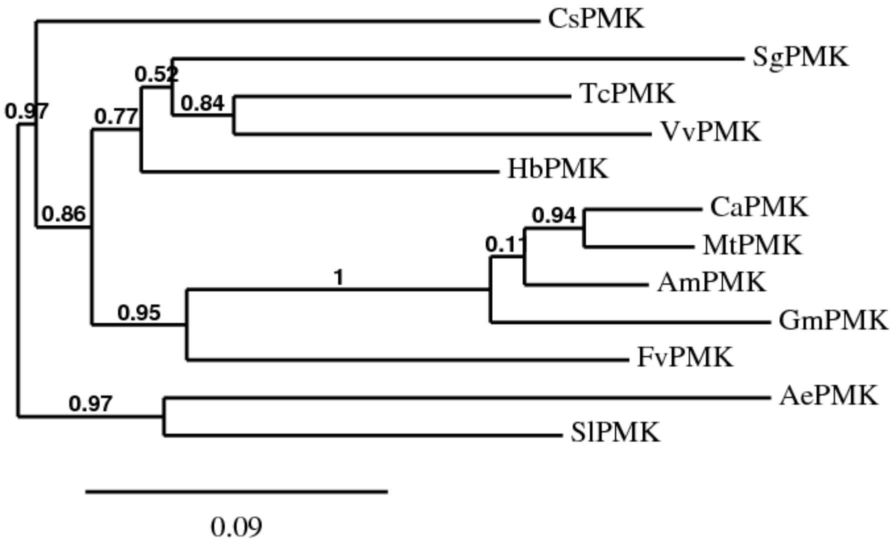

Figure S14. Phylogenetic tree of MVDs. *C. arietinum* (XP_004497159), *G. max* (XP_003555870), *C. roseus* (ADR65113), *R. communis* (XP_002521172), *F. vesca* (XP_004307061), *A. membranaceus* (KF355964), *C. arietinum* (XP_004497159), *H. brasiliensis* (BAF98285), *V. vinifera* (XP_002266399), *S. lycopersicum* (NP_001234815), *Arnebia euchroma* (ABG24207), and *A. thaliana* (NP_566995).

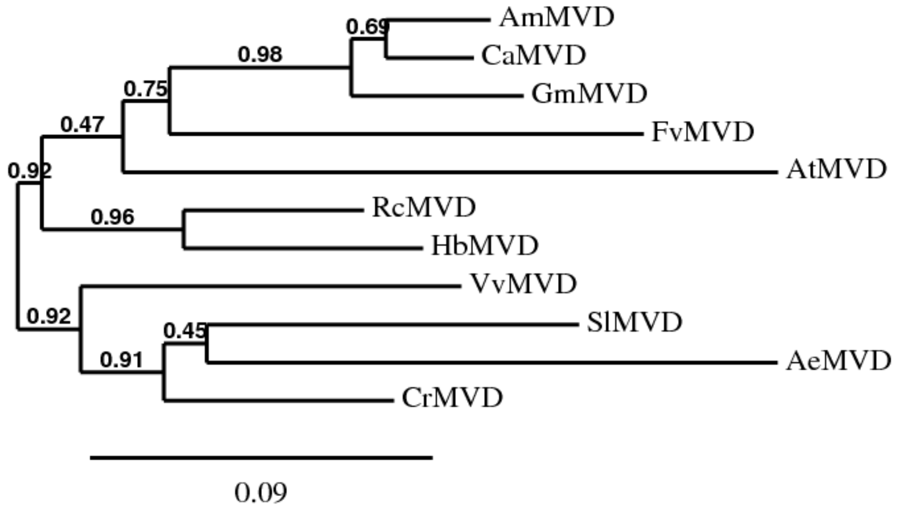

Figure S15. Phylogenetic tree of PMKs. *C. arietinum* (XP_004502634), *M. truncatula* (XP_003602220), *G. max* (XP_003526704), *V. vinifera* (XP_002275808), *F. vesca* (XP_004300184), *A. membranaceus* (KF355962), *T. cacao* (EOY25236), *S. lycopersicum* (XP_004241759), *C, sativus* (XP_004136210), *H. brasiliensis* (AAL18926), *S. grosvenorii* (AEM42973), and *Arnebia euchroma* (ABY27562).

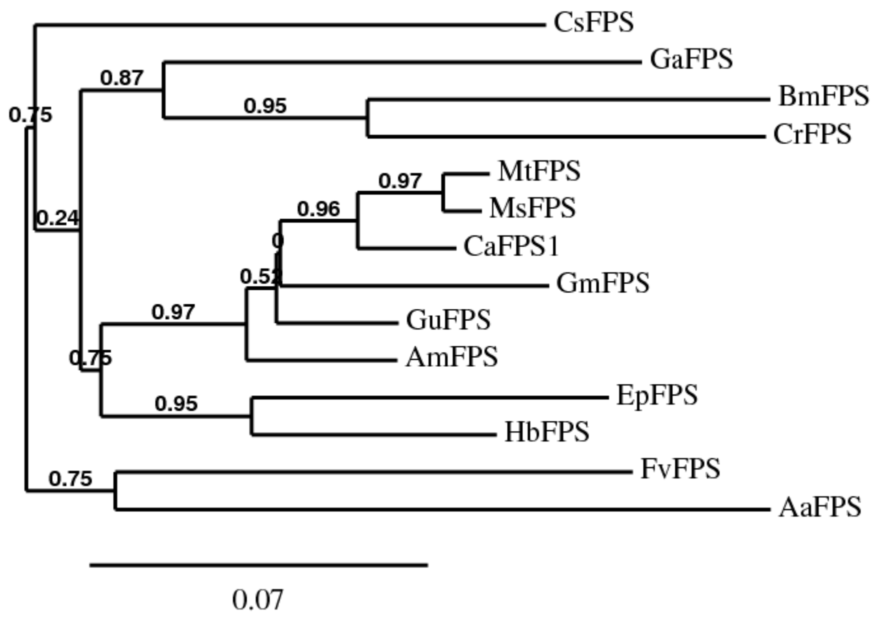

Supplement: Supplementary File 1 [file molecules-19-10922-s001.pdf]
